# Non-heparinized veno-venous ECMO applied to trauma patient with severe hypovolemic shock and hypothermia: A case report

**DOI:** 10.1097/MD.0000000000045837

**Published:** 2025-11-14

**Authors:** Seong Ho Moon, Jong Woo Kim, Dong Hoon Kang, Sang Kwon Lee, Ho Jeong Cha, Joung Hun Byun

**Affiliations:** a Department of Thoracic and Cardiovascular Surgery, Gyeongsang National University College of Medicine and Gyeongsang National University Changwon Hospital, Changwon-si, Republic of Korea.

**Keywords:** extracorporeal membrane oxygenation, hemorrhage, hypothermia, trauma

## Abstract

**Rationale:**

Extracorporeal membrane oxygenation (ECMO) can provide life-saving circulatory and thermal support in patients with severe trauma complicated by massive hemorrhage, conditions where conventional resuscitation may be insufficient. This report presents a case demonstrating that heparin-free veno-venous ECMO can be safely implemented in selected trauma patients to stabilize hemodynamics, restore normothermia, and facilitate definitive surgical management.

**Patient concerns:**

We report the case of a 26-year-old male who sustained multiple traumatic injuries after a traffic accident, most notably a left above-knee amputation that resulted in massive hemorrhage, along with minor right-toe fracture. The patient’s hemodynamic status was unstable due to excessive hemorrhage and hypothermia.

**Diagnoses:**

Severe traumatic hemorrhage with hypothermia following left above-knee amputation.

**Interventions:**

To enable aggressive resuscitation, heparin-free veno-venous ECMO (VV ECMO) was initiated peripherally via both femoral veins. ECMO provided circulatory and thermal support, allowing massive transfusion, restoration of normothermia, and stabilization for subsequent orthopedic surgery.

**Outcomes:**

ECMO enabled restoration of normothermia and hemodynamic stabilization, allowing subsequent orthopedic surgery for definitive bleeding control. The patient was weaned from mechanical ventilation on postoperative day 21, underwent tracheal decannulation on day 33, and was discharged on postoperative day 170 in a stable condition with full cognitive recovery.

**Lessons:**

In patients with severe trauma and hemorrhage, the application of ECMO, provided that clinically viable bleeding control is achieved, is thought to facilitate effective blood transfusion and help prevent hypothermia caused by massive hemorrhage and transfusion.

## 1. Introduction

Extracorporeal membrane oxygenation (ECMO) is generally used to treat refractory acute respiratory or cardiac failures. The traditional contraindication of ECMO in patients with bleeding due to the need for anticoagulation is being challenged by growing evidence that its hemodynamic support can improve patient outcomes, even trauma or pulmonary hemorrhage cases.^[1]^ Single-center series and observational reports have documented successful ECMO use in trauma patients with severe respiratory failure or circulatory compromise, even in patients with traumatic brain injury or hemorrhagic shock, provided that patient selection is rigorous and bleeding risk is at least partially manageable.^[2]^ Heparin-free ECMO may offer a promising avenue for addressing the challenges associated with systemic anticoagulation in ECMO therapy.^[3]^ Here, we report a case of successful ECMO application in a 26-year-old male patient who developed hypovolemic shock and hypothermia due to severe trauma with excessive bleeding.

## 2. Case presentation

A 26-year-old male presented to the emergency department 20 mins after a traffic accident with multiple traumatic injuries, including open fractures of the right toes and a left above-knee amputation. On admission, he was alert but in profound shock, with a blood pressure of 60/40 mm Hg, heart rate of 170 beats per minute, respiratory rate of 30 breaths per minute, and a body temperature of 32 °C. Laboratory findings revealed severe anemia with a hemoglobin level of 5 g/dL and thrombocytopenia with a platelet count of 62,000/μL. Arterial blood gas analysis showed metabolic acidosis (pH 7.20), hypoxemia (partial pressure of oxygen 100 mm Hg, saturated oxygen, 96.9%), and hypocapnia (carbon dioxide, 22 mm Hg). Coagulation studies demonstrated a prothrombin time-international normalized ratio of 1.41 and an activated partial thromboplastin time of 44.5 seconds.

**Figure 1.**
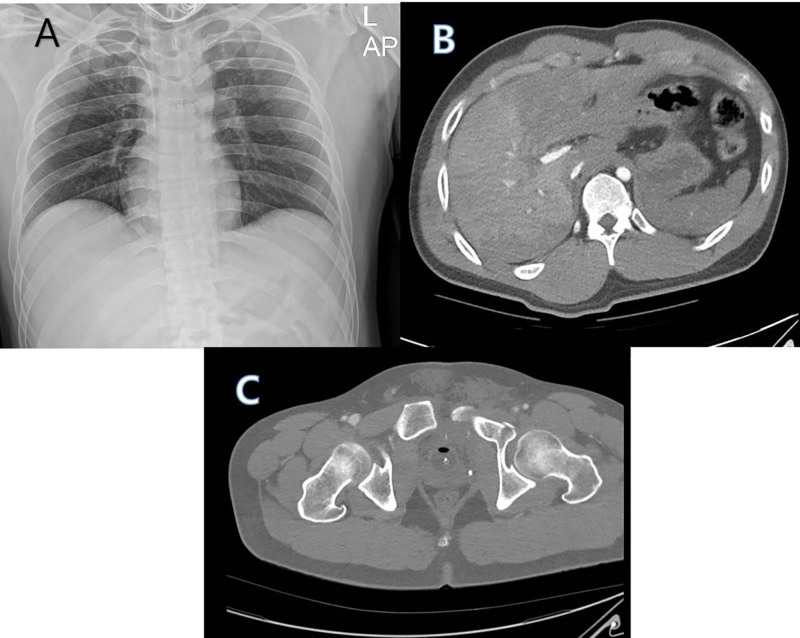
Simple chest radiography (A) shows no specific findings. Abdominal computed tomography (B and C) showing pelvic and sacral fractures, a hematoma in the pre-vesical space, and liver contusion.

**Figure 2.**
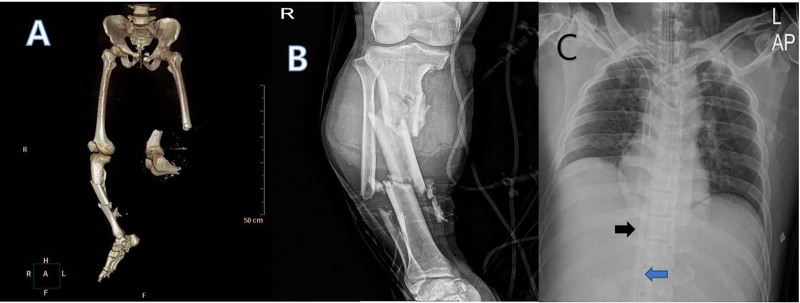
Three-dimensional computed tomography and simple radiography of legs shows amputation state of the left lower extremity (A) and fracture of the right tibia, right fibula, and right calcaneus (B). Veno-venous extracorporeal membrane oxygenation was applied peripherally through both femoral veins using a 21 (black arrow), 23 Fr (blue arrow) venous cannula (C).

Within 10 minutes of arrival, the patient experienced cardiac arrest. Cardiopulmonary resuscitation was performed for 2 minutes, after which spontaneous circulation was restored. He was intubated, and echocardiography confirmed preserved cardiac function. Simple chest radiography (Fig. 1A) and brain computed tomography showed no specific findings, and abdominal computed tomography (Fig 1B and C) showed pelvic and sacral fractures, a hematoma in the pre-vesical space, and a liver contusion. Figure 2A and B show the amputation state of the left lower extremity and fractures of the right tibia, right fibula, and right calcaneus. Given persistent hypovolemic shock with hypothermia, heparin-free veno-venous ECMO (MAQUET Cardiopulmonary AG, Hirrlingen, Germany) was initiated peripherally via bilateral femoral veins at a flow rate of 3.0 L/min, approximately 1 hour after arrival (Fig. 2C).

Normothermia was rapidly achieved using a heat exchanger (HU 35 Heater Unit), and massive transfusion was performed through the ECMO circuit, consisting of 15 units of packed red blood cells and 10 units of fresh frozen plasma. The patient’s blood pressure and cardiac rhythm stabilized sufficiently to permit definitive orthopedic surgery.

Approximately 90 minutes after admission, orthopedic surgery with bilateral above-knee amputation was undertaken while ECMO provided stable oxygenation (saturated oxygen around 95%) and protective ventilation (FiO2: 0.5, PEEP: 5 cmH2O, PIP: 24 cmH2O). Intraoperatively, body temperature was maintained at 37 °C and effective blood product delivery was achieved.

Over the next 12 hours, despite initial hemodynamic stabilization, fluctuating hemoglobin levels required additional transfusion of 6 units of red blood cells and 8 units of platelets. Angiography subsequently revealed multiple punctate bleeding foci around the left pubic bone, which were controlled by embolization. With bleeding stabilized and vital signs normalized, ECMO was successfully weaned 24 hours after initiation. After ECMO removal, the patient demonstrated gradual improvement in respiratory function and was successfully weaned from mechanical ventilation on postoperative day 21 following tracheostomy. Tracheal decannulation was performed on postoperative day 33, and the patient began active rehabilitation. No thrombotic or major bleeding complications related to ECMO were observed. The patient was discharged on postoperative day 170 in a stable condition and continues regular outpatient follow-up without respiratory or circulatory sequelae.

## 3. Discussion

Clinical outcomes in trauma patients are positively influenced by therapeutic modalities such as fluid resuscitation, mechanical ventilation, and related procedures. Severe hemorrhagic shock is associated with poor clinical outcomes. Optimal patient outcomes in severe trauma complicated by hemorrhagic shock necessitate treatment focused on restoring circulatory stability through targeted transfusion, rapid hemorrhage control, and sustained normothermia.^[4]^ In cases of severe trauma, pulmonary edema may develop or worsen after hemorrhagic shock as a result of transfusion-associated acute lung injury or circulatory overload secondary to aggressive crystalloid resuscitation. As highlighted by Flatley et al (2024), ECMO can serve as a supportive strategy in such scenarios by providing adequate oxygenation and hemodynamic stability while mitigating the risk of ventilator-induced or fluid-overload–related lung injury.^[5]^ In our patient, ECMO was initiated not only to maintain normothermia and enable massive transfusion but also to preemptively address the heightened risk of acute pulmonary edema, thereby facilitating safe progression to definitive surgical management.

In severe hypothermia, the cardiovascular system undergoes critical pathophysiological changes, characterized by the onset of cardiac dysrhythmias, a marked decrease in cardiac output, a reduction in central nervous system electrical activity, and cold-induced diuresis, with potential for noncardiogenic pulmonary edema.^[6]^

A rare but illustrative report described rhabdomyolysis as a complication of therapeutic hypothermia after cardiopulmonary resuscitation,^[7]^ underscoring the dangers of uncorrected hypothermia.

We initiated heparin-free VV ECMO due to the high risk of acute pulmonary edema or lung injury from massive blood transfusion and the significantly increased risk of exacerbating hypothermia and acidosis. The risk of circuit thrombosis during heparin-free ECMO is well recognized. As demonstrated by Fina et al., ECMO can be successfully performed without systemic anticoagulation in selected patients, although meticulous monitoring for thrombotic complications remains essential.^[8]^

Clinically, it is crucial to correct the so-called “triad of death” (acidosis, hypothermia, and coagulopathy) to ensure patient survival. One method of overcoming these conditions is ECMO (Extracorporeal Membrane Oxygenation).^[9]^

Another important issue is anticoagulation. While most ECMO patients require systemic heparinization, this poses major risks in trauma. Our case shows that heparin-free ECMO can be feasible when thrombotic risk is acceptable. Careful assessment of the patient’s coagulation status is critical, and risk tools such as the CHA₂DS₂-VASc score, though developed for cardiac disease, may also inform anticoagulation strategies and patient selection.^[10]^

Compared to prior reports, our case aligns with the growing body of evidence suggesting that ECMO can be beneficial in carefully selected trauma patients. For example, Arlt et al reported survival benefits in polytrauma patients with hemorrhagic shock when bleeding was controlled prior to ECMO initiation, while Willers et al demonstrated that ECMO is feasible in hemorrhagic conditions but stressed the importance of strict patient selection.^[1,11]^

However, important limitations and risks must be acknowledged. The use of ECMO in uncontrolled bleeding remains highly controversial. In such circumstances, the risk of circuit clotting, worsening coagulopathy, and difficulty in managing anticoagulation outweigh the potential benefits Some authors emphasize that systemic anticoagulation, or even ECMO circuit exposure, can exacerbate hemorrhage and coagulopathy, particularly in trauma patients with ongoing bleeding or coagulopathy.^[12]^ Thus, ECMO should not be regarded as a universal solution for all trauma patients.

VV ECMO was applied to the patient to restore oxygenation, maintain normothermia, and ensure massive transfusion and lung-protective ventilation during the operation. Without applying ECMO to the patients, they would not have adequately recovered from the hypovolemic state and would have struggled to maintain their body temperature. Consequently, the patient could have progressed to organ failure because of exacerbated metabolic acidosis.

This report has several limitations. As a single case study, the findings cannot be generalized to all trauma patients requiring extracorporeal support. The decision to initiate heparin-free ECMO was based on the specific clinical context, including the balance between bleeding risk and hemodynamic instability, which may vary considerably among patients.

In conclusion, ECMO in trauma should be considered only in highly selected patients, with careful assessment of bleeding control and physiologic reserve. This case illustrates that under these conditions, heparin-free VV ECMO can provide life-saving circulatory and thermal support, while its indiscriminate use in uncontrolled hemorrhage remains unsafe and potentially harmful.

## Author contributions

**Conceptualization:** Joung Hun Byun.

**Data curation:** Jong Woo Kim, Dong Hoon Kang, Sang Kwon Lee, Ho Jeong Cha.

**Formal analysis:** Seong Ho Moon, Joung Hun Byun.

**Methodology:** Seong Ho Moon, Joung Hun Byun.

**Supervision:** Joung Hun Byun.

**Writing – original draft:** Seong Ho Moon, Joung Hun Byun.

**Writing – review & editing:** Joung Hun Byun.
